# Vasa praevia with meandering fetal vessels despite placental and umbilical cord insertion on the same side of the uterine wall: A case report

**DOI:** 10.1002/ccr3.4142

**Published:** 2021-05-06

**Authors:** Tatsuro Horiuchi, Hiroshi Sato, Katsunori Matsui, Makiko Ikeda, Hajime Morishita, Masaya Hirose

**Affiliations:** ^1^ Department of Obstetrics and Gynecology Hyogo Prefectural Amagasaki General Medical Center Amagasaki Japan

**Keywords:** Cesarean section, placenta, prenatal diagnosis, ultrasound, vasa praevia

## Abstract

Vasa praevia with meandering fetal vessels is extremely rare, and it is difficult to diagnose this prenatally. When cesarean section is performed, a change in the site of uterine incision may be required for a safe delivery.

## Graphical abstract

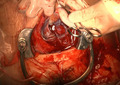

## 1 INTRODUCTION

A 38‐year‐old female, gravida 2, para 1, was referred to our hospital for perinatal management. She was diagnosed with vasa praevia with meandering fetal vessels at 37 weeks gestation. Emergency cesarean section was performed. Obstetricians should be familiar with vasa praevia with meandering fetal vessels despite its rare occurrence.

Vasa praevia is defined as a condition in which the cord vessels are present in the membranes covering or close to the internal cervical os. There are no standardized criteria specifying how close the fetal vessels must be to the internal os to constitute vasa praevia. A threshold of 2 cm has been proposed.^1^ In previous reports, the prevalence of vasa praevia was approximately 1 in 2500 pregnancies. In vitro fertilization (IVF) increases the risk of vasa praevia to approximately 1 in 250. Recent increases in pregnancy due to IVF may also increase the prevalence of vasa praevia. Two types of vasa praevia have been described. Type 1 occurs with a velamentous cord insertion, whereas Type 2 occurs with bilobed or succenturiate lobe placenta. In 90%–95% of these cases, vasa praevia is associated with placenta praevia, a low‐lying, bilobed, or succenturiate lobe placenta (Type 2), or with velamentous umbilical cord insertion (Type 1).^2^ An abnormal position and/or morphology of the placenta should prompt the physician to rule out vasa praevia. If the position and morphology of the placenta are normal on ultrasound examination, the possibility of fetal vessels running over the internal cervical os should be evaluated for a diagnosis of vasa praevia. In many cases of Type 2 vasa praevia, the placenta and/or site of cord insertion should be low‐lying. In such cases, fetal vessels usually run over the internal cervical os and continue to the main placenta on the opposite uterine wall. If the placenta and umbilical cord insertion are on the same side of the uterine wall, fetal vessels could potentially meander and run over the internal os. Such vasa praevia is extremely rare and difficult to diagnose prenatally. Herein, we report a case of vasa praevia with meandering fetal vessels despite placenta and umbilical cord insertion on the same side of the uterine wall.

## 2 CASE HISTORY/EXAMINATION

**FIGURE 1:**
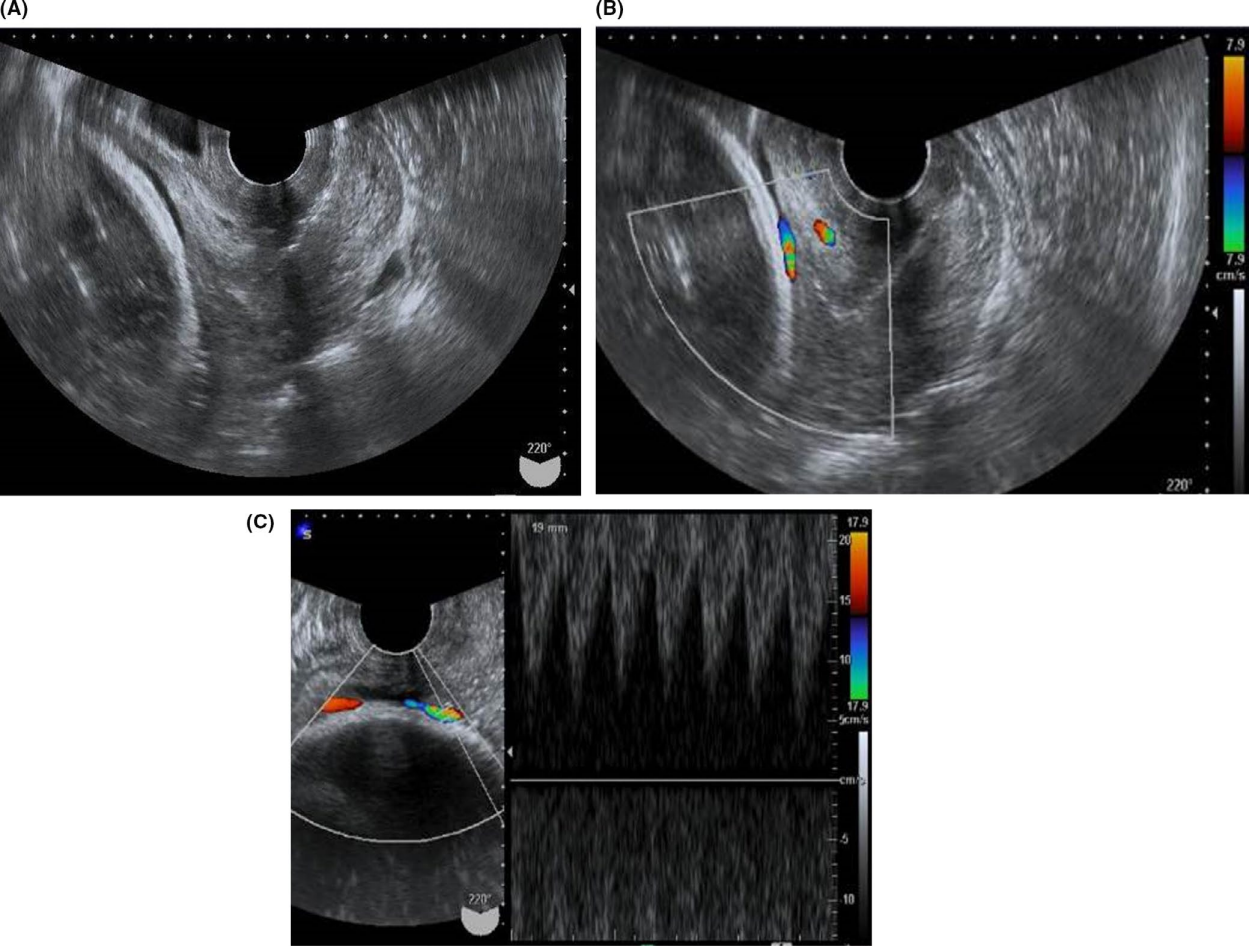
Transvaginal ultrasound without color Doppler did not reveal abnormal image such as the placenta and vessels between the fetal head and internal cervical os (A). A vessel was found between the fetal head and the internal cervical os on color Doppler (B). Using pulse Doppler, the vessel exhibited fetal artery waveforms (C)

A 38‐year‐old female, gravida 2, para 1, was referred to our hospital at 9 weeks gestation after *IVF*. Her past history included moyamoya disease. Delivery with epidural anesthesia has been recommended by the neurosurgeon. Her family history indicated that her father had died of moyamoya disease. At the second‐trimester screening, the placenta was attached to the anterior uterine wall. A low‐lying placenta, succenturiate lobe, and/or multilobed placenta were not identified. The umbilical cord insertion was marginal. The site of the umbilical cord insertion was on the lower side of the placenta. However, the placental position and the site of umbilical cord insertion were above the upper end of the bladder. At that time, vasa praevia was not suspected. At 37 weeks gestation, gestational hypertension occurred. Elective abortion was required due to gestational hypertension. Transvaginal ultrasound without color Doppler did not reveal an abnormal image between the fetal head and the internal cervical os (Figure 1A). However, ultrasound with color Doppler was performed to exclude abnormal insertion of the umbilical cord before the introduction of a transcervical balloon catheter for cervical ripening. A few fetal vessels were observed between the fetal head and the internal cervical os using color and pulse Doppler (Figure 1B, C).

### 2.1 Differential diagnosis, investigations, and treatment

**FIGURE 2:**
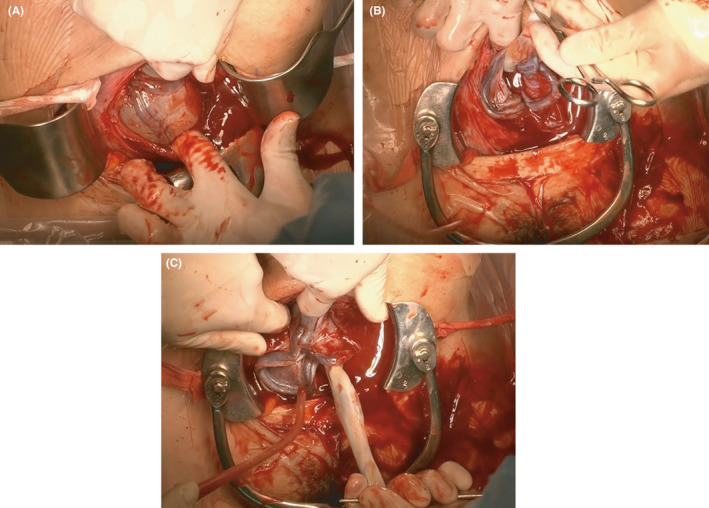
Intraoperative findings before rupture of the amniotic membrane indicated that the fetal vessels were found in the exposed amniotic membrane (A). A segment of the fetal vessels was meandering and running over the internal cervical os (B). Both the placental position and site of the umbilical cord insertion were above the low transverse incision scar (C). In all photographs, the upper side of the image was the side of the patient's head

**FIGURE 3:**
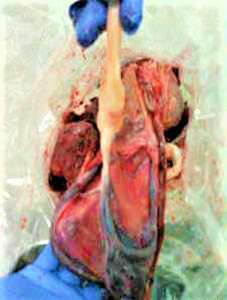
Examination of the placenta after delivery revealed that a part of the membranous fetal vessels (the lower side of the figure) was meandering far from the placenta and site of cord insertion (the upper side of the figure)

We diagnosed this patient with vasa praevia, and emergency cesarean section was performed. We predicted that the fetal vessels were running on the anterior wall of the lower uterine segment. Low transverse incision was performed. A careful blunt entry using hemostats and fingertips was performed to avoid fetal vessel injury prior to delivery. The amniotic membrane was exposed without rupture of the membrane and injury to the fetal vessels. Fetal vessels were found in the exposed amniotic membrane (Figure 2A). Because the vessels were running on both sides of the membrane, the amniotic membrane ruptured at the central part. A female baby weighing 2890 g was delivered without injury to the cord vessels. The Apgar scores were 8 and 9 at 1 and 5 min, respectively. We examined the intrauterine findings after delivery. As we diagnosed before the operation, a section of the fetal vessels was meandering and running over the internal cervical os (Figure 2B). Both the placental position and site of umbilical cord insertion were above the low transverse incision scar (Figure 2C). Examination of the placenta after delivery revealed a part of the membranous fetal vessels which were meandering far from the placenta and site of cord insertion (Figure 3).

### 2.2 Outcome and follow‐up

The postoperative course was uneventful.

## 3 DISCUSSION

The purpose of this case report was to draw the attention of the obstetrical practitioner to the importance of detailed transvaginal ultrasound examination including color Doppler to prevent accidents due to unusual vasa praevia rupture.

If vasa praevia is not diagnosed prenatally, fetal vessel rupture may occur, resulting in fetal hemorrhage. Oyelese et al reported that the neonatal survival rates for prenatally undiagnosed versus diagnosed vasa praevia are 43.6% and 97.6%, respectively.^3^ Therefore, prenatal diagnosis of vasa praevia is essential for a safe delivery.

The guideline of the International Society of Ultrasound and Gynaecology (ISUOG) for the second‐trimester examination mentions the placental position.^4^ If the lower placental edge reaches or overlaps the internal os, a follow‐up examination in the third trimester is recommended. The guideline mentions that detailed examination for vasa praevia may not be required if the position and morphology of the placenta are normal.

Prince reported a case of vasa praevia with anomalous umbilical cord formation.^5^ In this case, vasa praevia was not diagnosed prenatally. Routine screening by transabdominal ultrasound in the second and third trimester revealed an anterior placenta not covering the cervical os. Further examination for vasa praevia was not performed. Fortunately, vaginal delivery was possible without serious complications for the neonate. This case is similar to our case, wherein having an unexpected course of the fetal vessels resulted in the occurrence of vasa praevia. In such cases, transvaginal ultrasound without color Doppler may not be able to detect vasa praevia.

The accuracy of transvaginal ultrasound in the diagnosis of vasa praevia was previously reported to be high when performed in combination with color Doppler.^6^ Obstetricians usually perform transvaginal ultrasound without color Doppler. If the placenta and/or umbilical cord vessels are not detected near the internal cervical os on ultrasound without color Doppler, further examinations with color Doppler may not be conducted. In our case, ultrasound with color Doppler was performed to exclude abnormal insertion of the umbilical cord prior to introduction of a transcervical Foley catheter for cervical ripening. A few fetal vessels indicating vasa praevia were only detected after performing ultrasound with color Doppler, an incidental and fortunate diagnosis.

After making a diagnosis of vasa praevia, cesarean section before rupture of the fetal vessels is thought to be required for good prognosis of the neonate. If the fetal vessels are running on the anterior lower segment of the uterus, obstetricians should be careful to prevent intraoperative rupture of the fetal vessels. Aoki et al reported a case of vasa praevia in which the cord vessels were running on the anterior lower uterine segment.^7^ They made a vertical uterine incision to avoid rupture of the cord. In future pregnancies after making a vertical uterine incision, the risk of uterine rupture or placenta accreta is higher relative to that of an incision in the lower uterine segment. Increased rates of uterine rupture (4%–9% in case of trial of labor; around 1% in elective cesarean delivery, around 1%) and placenta accreta spectrum (around 1%) have been reported.^8^, ^9^ However, in their case, they concluded that an incision in the lower uterine segment would have carried a higher risk of rupture of the cord vessels and loss of massive fetal blood. We also predicted that the cord vessels were running on the anterior lower uterine segment in our case. Both the placental position and site of umbilical cord insertion were above the low transverse incision scar. A few fetal vessels were meandering and running over the internal cervical os. We thought that safe delivery *via* low transverse incision was feasible by putting the fetal vessels at the lateral part of the incision scar. If the membranous fetal vessels were shorter and/or the placenta was near the low transverse incision, it is difficult to create a sufficient space for a safe delivery. The precise diagnosis of vasa praevia and assessment of the course of cord vessels are essential for a safe delivery. A change in the site of uterine incision may be required. If a normal low transverse incision is not adopted, an increased risk in future pregnancies should be considered.

## CONFLICT OF INTEREST

All authors declare no conflict of interests.

## AUTHOR CONTRIBUTIONS

TH, HS, and MH: designed the research study. TH and HS: performed the research. TH and HS: analyzed the data. TH, HS, and MH: wrote the manuscript. All authors contributed to editorial changes in the manuscript. All authors read and approved the final manuscript.

## ETHICAL STATEMENT

Published with written consent of the patient.

## Data Availability

The data that support the findings of this study are available from the corresponding author upon reasonable request.
